# Inhibition of SARS CoV Envelope Protein by Flavonoids and Classical Viroporin Inhibitors

**DOI:** 10.3389/fmicb.2021.692423

**Published:** 2021-07-08

**Authors:** Ulrike Breitinger, Nourhan K. M. Ali, Heinrich Sticht, Hans-Georg Breitinger

**Affiliations:** ^1^Department of Biochemistry, German University in Cairo, New Cairo, Egypt; ^2^Division of Bioinformatics, Institute of Biochemistry, Friedrich-Alexander-Universität Erlangen-Nürnberg, Erlangen, Germany

**Keywords:** SARS CoV-1, E protein, viroporins, cell viability assay, patch-clamp electrophysiology, ion channel inhibitors

## Abstract

Severe acute respiratory syndrome coronavirus (SARS-CoV), an enveloped single-stranded positive-sense RNA virus, is a member of the genus *Betacoronavirus*, family Coronaviridae. The SARS-CoV envelope protein E is a small (∼8.4 kDa) channel-forming membrane protein whose sequence is highly conserved between SARS-CoV and SARS-CoV-2. As a viroporin, it is involved in various aspects of the virus life cycle including assembly, budding, envelope formation, virus release, and inflammasome activation. Here, SARS-CoV E protein was recombinantly expressed in HEK293 cells and channel activity and the effects of viroporin inhibitors studied using patch-clamp electrophysiology and a cell viability assay. We introduced a membrane-directing signal peptide to ensure transfer of recombinant E protein to the plasma membrane. E protein expression induced transmembrane currents that were blocked by various inhibitors. In an ion-reduced buffer system, currents were proton-dependent and blocked by viroporin inhibitors rimantadine and amantadine. I-V relationships of recombinant E protein were not pH-dependent in a classical buffer system with high extracellular Na^+^ and high intracellular K^+^. E-protein mediated currents were inhibited by amantadine and rimantadine, as well as 5-(N,N-hexamethylene)amiloride (HMA). We tested a total of 10 flavonoids, finding inhibitory activity of varying potency. Epigallocatechin and quercetin were most effective, with IC_50_ values of 1.5 ± 0.1 and 3.7 ± 0.2 nM, respectively, similar to the potency of rimantadine (IC_50_ = 1.7 ± 0.6 nM). Patch-clamp results were independently verified using a modified cell viability assay for viroporin inhibitors. These results contribute to the development of novel antiviral drugs that suppress virus activity and proliferation.

## Introduction

Coronaviruses (CoVs) cause respiratory diseases in humans ranging from common colds, bronchiolitis to acute respiratory distress syndrome (ARDS) and fatal pneumonias. Severe acute respiratory syndrome coronavirus (SARS-CoV) and SARS-CoV-2 are CoVs related to severe acute respiratory syndrome (SARS), belonging to the order Nidovirales, family Coronaviridae, genus Betacoronavirus, subgenus Sarbecovirus, are enveloped, single-stranded, positive sense RNA viruses with a genome of about 30 kb (Gonzalez et al., 2003). SARS-CoV-2, SARS-CoV and MERS-CoV (subgenus Merbecovirus) are highly pathogenic zoonotic viruses; the associated diseases are COVID-19 (2019 Wuhan, China), SARS (2002 Guangdong province, China) and MERS (2012 Kingdom of Saudi Arabia) whose mortality severely affected the economies of countries where the viruses spread (Liu et al., 2020). At the end of 2002, SARS CoV emerged in Guandong province, Southeast China, initiating a global epidemic (Nieto-Torres et al., 2015). The virus had a mortality rate of 10% (Drosten et al., 2003), and infected approximately 8000 people. SARS-CoV-2 was first observed in November 2019 in Wuhan, China. After a rapid worldwide spread, many countries have presently developed a second, some even a third wave of virus infection. Severe cases result in ARDS with systemic inflammation; lung injury is associated with release of inflammatory cytokines interleukin-6 (IL-6) and IL-1b (Freeman and Swartz, 2020). Release of inflammatory cytokines in hepatitis C (Shrivastava et al., 2013; Farag et al., 2017, 2020; Negash et al., 2019) is associated with ion channel activity of the HCV p7 protein (Farag et al., 2017, 2020). COVID-19 severity is associated with increased proinflammatory cytokines and chemokines and IL-6, specifically, is predictive of COVID-19 fatality (Costela-Ruiz et al., 2020).

The single positive-strand RNA genome of SARS CoV contains 14 open reading frames (ORFs) encoding structural proteins, such as spike protein (S), membrane protein (M), envelope protein (E), and nucleocapsid protein (N), as well as viral replicase and protease (Marra et al., 2003). The envelope protein E belongs to the group of viroporins. These types of proteins are mostly small, hydrophobic, integral membrane proteins that homo-oligomerize to form membrane channels in host cell membranes. In 1992, the first viroporin, the M2 channel of the influenza A Virus, was discovered and studied (Duff and Ashley, 1992; Pinto et al., 1992). Since then, several viroporins have been identified and investigated in other pathogenic viruses, including the p7 ion channel of Hepatitis C virus (HCV) (Griffin et al., 2003; Pavlovic et al., 2003; Breitinger et al., 2016; Farag et al., 2017), the Vpu ion channel of human immunodeficiency virus (HIV)-1 (Ewart et al., 1996), as well as proteins E (Wilson et al., 2004; Verdia-Baguena et al., 2012) and 3A of CoVs (Lu et al., 2006). Viroporins control several steps of the virus replication cycle, including entry, genome replication, morphogenesis, and release from the infected cell (Hover et al., 2017). Several viroporins play important roles in viral pathogenesis, promoting ion imbalances within cells (Zhang et al., 2013) or disrupting cellular pathways through protein-protein interactions (Andrew and Strebel, 2010). Viroporins possess great potential as antiviral targets leading to substantial interest in the study of these proteins (Hover et al., 2017).

Several CoVs, such as MERS-CoV, HCoV-229E, HCoV-OC43, and porcine epidemic diarrhea virus (PEDV) encode two viroporins. SARS-CoV, on the other hand, encodes three viroporins, namely 3a, E, and 8a (Castano-Rodriguez et al., 2018). While the full-length E and 3a proteins are required for maximal SARS-CoV replication and virulence, viroporin 8a has only a minor impact on these activities. E protein ion channel activity and the presence of its PDZ-binding motif (PBM) are necessary for virulence in mice. The presence or absence of the homologous motifs in protein 3a do not influence virus pathogenicity, thus ion channel activity and PBM of the E protein are dominant over those of protein 3a (Castano-Rodriguez et al., 2018).

**FIGURE 1:**
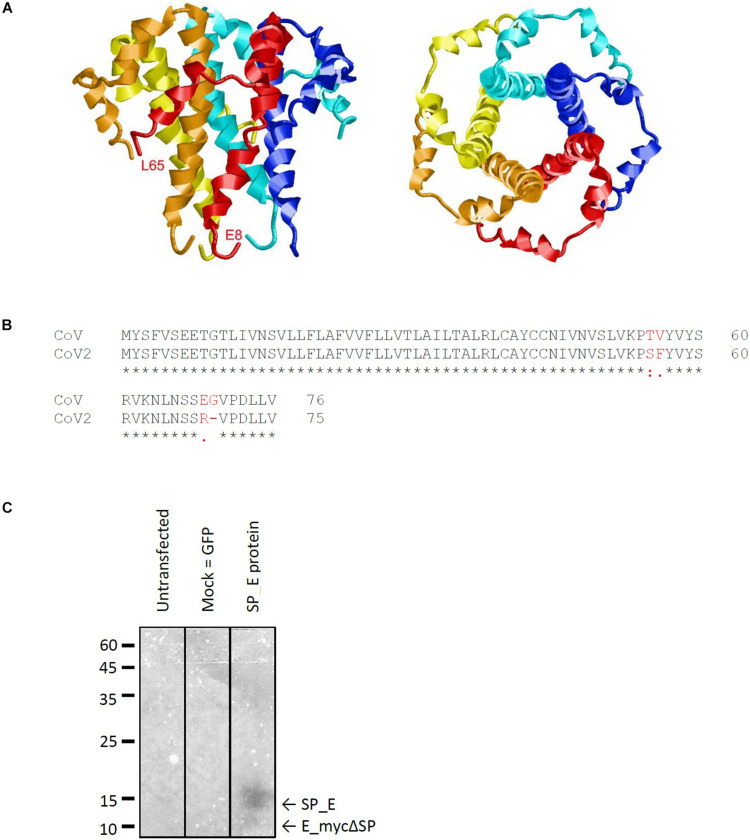
Characteristics of the SARS CoV E protein. **(A)** Structural model of the E protein (PDB code: 5X29) determined by NMR Spectroscopy in lyso-myristoyl phosphatidyl-glycerol (LMPG) micelles (Surya et al., 2018). The five subunits are shown in different colors and the N- and C-terminal residues resolved in the structure are labeled for one subunit. **(B)** Sequence alignment of the E protein of SARS against that of SARS CoV-2 reveals four amino acid exchanges. **(C)** Western blot analysis of recombinantly expressed SARS CoV E protein including a membrane directed signal peptide (SP_E). The theoretical sizes of E protein with and without signal sequence are 16.37 kDa (SP_E) and 12.4 kDa (E_mycΔSP), respectively.

The E protein of CoVs is a small transmembrane protein of ∼76 to 109 amino acids in length (Arbely et al., 2004). Its amino acid sequence is quite divergent among different CoVs; nevertheless, its structure (Surya et al., 2018) is highly conserved and includes a short N-terminal amino acid stretch, an alpha helical transmembrane domain and an extramembraneous carboxyl terminal region (Figure 1A). The genomes of SARS-CoV and SARS-CoV-2 show a high level of overall similarity, having an identity of 79.6% (Liu et al., 2020). Of the three viroporins – E, 3a and 8a –, the sequence of the E protein of SARS-CoV and SARS-CoV-2 is particularly conserved: Only four out of 76 amino acids are different between the two virus strains (Figure 1B). The other two viroporins are less conserved; 3a possesses an identity of 72.4% between SARS-CoV and SARS-CoV-2, while 8a is least conserved, with only 17.4% identity remaining between SARS-CoV and SARS-CoV-2.

Severe acute respiratory syndrome E protein mainly distributes between ER and Golgi apparatus membranes where it actively participates in virus budding, morphogenesis and trafficking (Verdia-Baguena et al., 2012). The protein localizes particularly to the endoplasmic reticulum-Golgi intermediate compartment (ERGIC) causing epithelial disruption which contributes to lung damage observed in SARS patients (Verdia-Baguena et al., 2012; Li et al., 2014). SARS E protein was also hypothesized to be present at the cell plasma membrane (Pervushin et al., 2009), yet this could not be confirmed in later studies (Nieto-Torres et al., 2011). CoV E protein forms oligomeric structures building an ion channel pore in planar lipid bilayers and micelles (Wilson et al., 2004; Torres et al., 2006; Pervushin et al., 2009). The E protein of SARS-CoV possesses ion channel activity for monovalent cations, with a 10-fold preference for sodium over potassium ions (Wilson et al., 2004). Substitution of Asn15 by Ala in a lysine-flanked peptide of the hydrophobic domain of SARS-CoV E protein abolished its channel conductance, predicting that Asn15 is essential for oligomerization or ion conductance (Ruch and Machamer, 2012). Deletion of the E gene in different CoVs leads to reduced virus maturation and release, production of low-virulence virus, and a reduction of cellular stress and virus-induced apoptosis (Ruch and Machamer, 2012).

The therapeutic potential of viroporins as antiviral targets was demonstrated by the identification of several small compounds that could successfully block or interfere with the ion channel activity of this class of proteins, which makes them an ideal target for therapeutic intervention (Nieva et al., 2012; Scott and Griffin, 2015). Classic inhibitors are the group of adamantanes – amantadine and rimantadine (Torres et al., 2007; Jing et al., 2008), 5-(N,N-hexamethylene)amiloride (HMA) (Wilson et al., 2006; Pervushin et al., 2009; Jalily et al., 2016), and several iminosugar derivatives (Pavlovic et al., 2003; Breitinger et al., 2021).

Patch-clamp electrophysiology is the method of choice to study the function of ion channels as it allows the direct measurements of channel-induced currents. The study of transiently expressed ion channels in HEK293 cells by whole-cell patch-clamp requires channels to be present in the plasma membrane. Ion channel proteins such as neuronal receptors usually contain a signal sequence that directs them to the plasma membrane. In contrast, viroporin activity is intracellular, therefore, these channels lack such a signal sequence. Indeed, CoVs, like the SARS-CoV, bud from the ERGIC. E protein was shown to co-locate with endoplasmic reticulum markers in perinuclear patches (Nal et al., 2005). In immune-fluorescent staining and cell fractionation studies the SARS CoV E protein is mainly found to be localized to the ER and the Golgi apparatus. However, a certain proportion of protein can be translocated to the cell surface where it is partially associated with lipid rafts (Liao et al., 2006). Pervushin et al. (2009) observed channel activity in HEK293 cells, transiently expressing full length SARS-CoV E protein in a whole-cell patch clamp set-up. This would be in agreement with E protein partly be localized at the cell surface. However, another study found the E protein mainly located in the ERGIC of cells that were transfected with a plasmid encoding E protein, or directly infected with SARS-CoV (Nieto-Torres et al., 2011). In this study, E protein could not be detected in the plasma membrane by immunofluorescence, immunoelectron microscopy, or cell surface protein labeling (Nieto-Torres et al., 2011). Furthermore, measurement of plasma membrane voltage gated ion channel activity by whole-cell patch clamp also suggested that E protein was not present in the plasma membrane (Nieto-Torres et al., 2011). A topology was proposed for the E protein where the amino terminus is oriented toward the lumen of intracellular membranes and carboxy terminus faces the cytoplasm (Nieto-Torres et al., 2011), which may hinder plasma membrane transport, or render the E protein inactive. In our work, we introduced a plasma membrane-directing signal sequence N-terminal to the E protein to ensure delivery to the plasma membrane.

Here, we tested inhibition of the E protein of the SARS coronavirus by classical viroporin inhibitors amantadine, rimantadine and HMA, and a series of plant metabolites including the flavonols quercetin and kaempferol, the flavones apigenin and nobiletin, the isoflavone genistein, naringenin, a flavanone, the catechin epigallocatechin gallate (EGCG), as well as the gingerols 6-gingerol and 8-gingerol, and the polyphenol resveratrol. A combination of a 3-[4,5-dimethylthiazole-2-yl]-2,5-diphenyltetrazolium bromide (MTT)-based cell viability assay and patch-clamp electrophysiology was used that had been previously established and tested for the p7 protein of hepatitis C (Pavlovic et al., 2003; Breitinger et al., 2021). We could confirm the ion channel activity of the E protein and identified rimantadine, HMA, quercetin and EGCG as promising inhibitors of the E protein channel.

## Materials and Methods

### Generation of SARS CoV E Protein Construct

E protein DNA was generated using an overlap extension PCR protocol and then ligated into the pRK5 vector using the restriction sites *Eco*RI and *Pst*I as described earlier (Breitinger et al., 2016).

### Cell Culture and Transfection

HEK293 cells (ATCC, LGC Standards GmbH, Wesel, Germany) were cultured in 10 cm tissue culture petri dishes in Minimum Essential Medium (EMEM, Sigma-Aldrich, Deisenhofen, Germany) supplemented with 10% FBS (Invitrogen, Karlsruhe, Germany) and Penicillin/Streptomycin (Sigma-Aldrich, Deisenhofen, Germany) at 5% CO_2_ and 37°C in a water-saturated atmosphere (Breitinger et al., 2016). For electrophysiological experiments, cells were plated on acetone treated glass coverslips in 24 well plates and transfected 1 day after passage using 1 μg of E protein cDNA, 1 μg of green fluorescence protein (GFP) cDNA and 3 μl polyethyleneimine (PEI) (Sigma-Aldrich, Deisenhofen, Germany) per well. We performed measurements 2–3 days after transfection.

### Western Blot Analysis

HEK293 cells were harvested 3 days after transfection, a crude membrane fraction was prepared and subjected to SDS-PAGE and Western blotting. An alkaline phosphatase-conjugated anti-c-myc antibody (Santa Cruz, Heidelberg, Germany) was used and the blot was visualized using 0.03% nitro blue tetrazolium and 0.02% 5-bromo-4-chloro-3-indolyl-phosphate in substrate buffer (100 mM Tris–HCl, pH 9.5; 100 mM NaCl; and 5 mM MgCl_2_).

### Cell Viability Assay

A total 120 μl of a HEK293 cell suspension were seeded to give ∼60,000 cells per 96-well plate well. Cells were transfected 1 day after plating using PEI. To this end, 0.3 μg of E protein or pRK5 vector (control) in 7 μl of EMEM were combined with 0.6 μl of PEI in 7 μl of EMEM, incubated for 20 min and added to each well. Next, 6.5 μl of inhibitor stock in EMEM were added to achieve final concentrations as required (usually between 1 and 100 μM). Four days after transfection, the MTT assay was performed as described before (Breitinger et al., 2021). The absorbance of formazan was taken in a Victor-3 plate reader (Perkin-Elmer, Berlin, Germany) at a wavelength of 595 nm. Formation of formazan requires active mitochondria and is a measure for the number of viable cells. Viability of cells transfected with E protein cloned into pRK5 vector were compared to cells transfected with empty pRK5 vector. Further controls included: (1) GFP transfected cells to verify efficient transfection; (2) the effect of transfection on cell viability was investigated by comparing untransfected cells to pRK5 transfected cells; (3) 200 mM KCl was added to untransfected cells to induce complete cell death, these readings were subtracted from data for background correction.

Normalized absorbance was calculated by subtracting background (200 mM KCl) and then dividing by the control absorbance from pRK transfected HEK293 cells. These control cells did not express E protein but were otherwise treated in the same way as E protein expressing cells (Supplementary Figure 1). Viability readings from E protein-expressing HEK293 cells in presence of varying concentrations of inhibitor (Supplementary Figure 1B, black columns) was divided by the reading from empty pRK vector-transfected control cells under same inhibitor concentration (Supplementary Figure 1B, gray columns). Control measurements using the empty expression vector (Supplementary Figure 1C) gave information about cytotoxic or proliferative effects of the compounds studied.

### Electrophysiological Recordings and Data Analysis

For experiments, cells were kept in a bathing solution containing 135 mM NaCl, 5.5 mM KCl, 2 mM CaCl_2_, 1.0 mM MgCl_2_, and 10 mM Hepes (pH 7.4 with NaOH). Current responses were measured at 21–23°C at a holding potential of −60 mV. Whole-cell recordings were performed using a HEKA EPC10 amplifier, controlled by Pulse software (HEKA Electronics, Lambrecht, Germany). Recording pipettes were pulled from borosilicate glass (World Precision Instruments, Berlin, Germany) using a Sutter P-97 horizontal puller (Sutter, Novato, CA, United States). Solutions were applied using an Octaflow system (NPI Electronics, Tamm, Germany), where cells were bathed in a laminar flow of buffer, giving a time resolution for solution exchange and re-equilibration of about 100 ms. The external buffer consisted of (in mM) 90 N-Methyl-D-glucamine (NMDG), 3 CaCl_2_, 90 HEPES and 90 2-(N-morpholino) ethane sulfonic acid (MES). The pH of the external buffer was adjusted to 7.5 and 5.5 using NaOH or HCl. Internal buffer was (in mM) 90 NMDG, 10 ethylene glycol tetraacetic acid (EGTA), 180 HEPES, pH 7.5 (NaOH). This technique has been described earlier (Breitinger et al., 2016) and was originally adapted from Chizhmakov et al. (2003). After setting the pH, buffers were salt-adjusted to ensure the same osmolarity at all pH values. For measurement of whole-cell E-protein-mediated currents, cells were perfused continuously with recording buffer during 1-min recording intervals. Inhibitors were diluted from stock solutions (10 mM) into pH 5.5 buffer. Inhibition measurements were normally started with baseline recordings of extracellular buffer pH 7.5 (4–5 s), followed by recordings at pH 5.5 (to induce channel opening). Then inhibitors (at pH 5.5) were applied, followed by final control applications. We perfused control solution (pH 5.5 without inhibitor) between different inhibitor concentrations. Typical current amplitudes were between 100 and 400 pA. Dose-response curves were constructed by averaging results from 4 to 5 cells, and inhibitor IC_50_ values were determined using a non-linear fit to the equation *I*_obs_ = *I*_max_/[1 + ([*I*]/IC_50_)]. *I*_obs_ is the observed current at any given concentration of inhibitor, *I*_max_ is the maximum current amplitude observed in the absence of inhibitor, and [*I*] is the concentration of inhibitor. For measurements using the current–voltage ramp method, we used a different combination or external and internal buffer. Here, external buffer consisted of (in mM) 135 NaCl, 5.5 KCl, 2 CaCl_2_, 1.0 MgCl_2_ and 10 Hepes (pH adjusted to 7.2 with NaOH); the internal buffer was (in mM)140 CsCl, 1.0 CaCl_2_, 2.0 MgCl_2_, 5.0 EGTA and 10 Hepes (pH adjusted to 7.2 with CsOH). Control cells were transfected with pRK5 vector co-transfected with GFP and compared to cells transfected with E protein and GFP. The applied voltage ramp ranged from −60 mV to +50 mV in 10 mV steps. For measurements in presence of inhibitor, we added channel blockers to the extracellular bath and a minimum of 6 cells were averaged.

## Results

**FIGURE 2:**
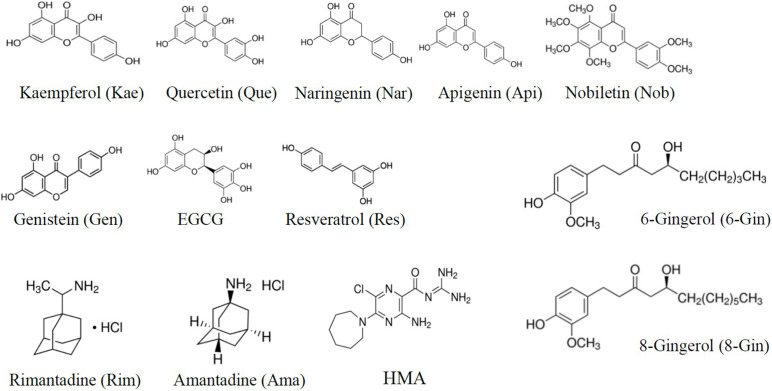
Structures of inhibitors used in this study. Substance classes were flavonols (quercetin, kaempferol), flavones (apigenin, nobiletin), isoflavones (genistein), flavanones (naringenin), gingerols (6-gingerol, 8-gingerol), polyphenols (resveratrol), and catechins (epigallocatechin gallate, EGCG), as well as the classical viroporin inhibitors amantadine, rimantadine, and 5-(N,N-hexamethylene) amiloride (HMA).

To investigate different channel blockers of SARS CoV E protein (Figure 1), we generated a cDNA construct by a primer walking protocol of consecutive PCR reactions (Breitinger et al., 2016). Using *Eco*RI and *Pst*I sites, the complete coding sequence was inserted into the pRK5 vector including a C-terminal myc-tag for Western blot analysis. E protein was expressed in HEK293 cells for Western blot analysis, MTT assays and electrophysiological measurements. Western blot analysis confirmed expression of the target protein (Figure 1C). The C-terminal myc-tag had no influence on ion conductivity, which was verified by patch clamp recordings. A range of known viroporin inhibitors as well as several flavonoids were tested for their activity against recombinant E protein. Investigated classical inhibitors were amantadine, rimantadine and HMA (Figure 2). Several flavonoids were reported in the literature for their antiviral activity. In most cases this effect is due to inhibition of SARS and MERS proteases 3CLpro and PLpro (Solnier and Fladerer, 2020). Genistein inhibits HIV ion channel viral protein U activity (Sauter et al., 2014) while Kaempferol blocks SARS CoV 3a channels (Schwarz et al., 2014). In this study, we investigated a total of 10 flavonoids for their potency as inhibitors of the E-protein channel. These included quercetin and kaempferol (flavonols), apigenin and nobiletin (flavones), genistein (isoflavone), naringenin (flavanone), the gingerols 6-gingerol and 8-gingerol, resveratrol (a polyphenol), and EGCG, a catechin (Figure 2).

Severe acute respiratory syndrome coronavirus E protein activity was shown to introduce cell stress and apoptosis (An et al., 1999; DeDiego et al., 2011). In a previous study, we exploited viroporin-mediated cytotoxicity as the basis for a new assay for inhibitors of the hepatitis C virus p7 channel (Breitinger et al., 2021). Comparison to several accepted p7 activity tests established the MTT cell viability assay of p7-transfected HEK293 cells as a quick and reliable test for the potency of viroporin inhibitors.

**FIGURE 3:**
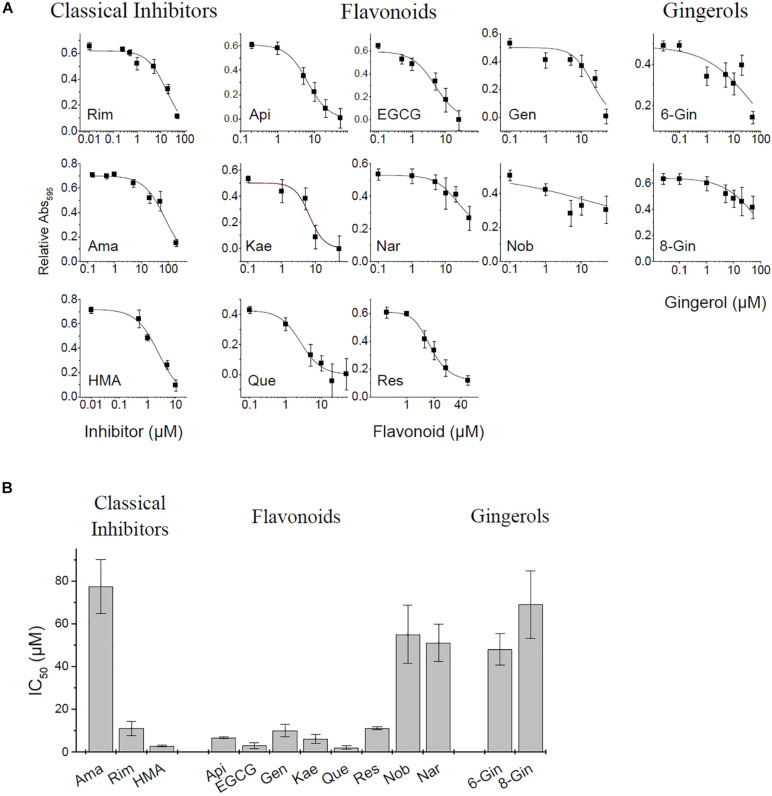
MTT Cell viability assay for E protein activity. **(A)** Normalized absorbance at different inhibitor concentration. See Supplementary Figure 1 for more detail on pRK control and E protein data used for analysis. **(B)** Estimated IC_50_ values of investigated inhibitors (means ± SEM).

In the present study we applied the MTT assay to test different channel blockers on SARS CoV E protein expressed in HEK293 cells. The cell viability assay is sensitive to both, viroporin-induced cell death as well as general cytotoxicity of test compounds. MTT cell viability measurements were performed using the empty pRK5 expression vector (control cells, no E protein) and E-protein expressing cells (Supplementary Figure 1). Readings were corrected for background (see section “Materials and Methods”). Control cells revealed cytotoxic or proliferative effects of the tested compounds. Viability readings from E-protein expressing cells were corrected for the readings form similarly treated control cells, revealing the extent of cell damage mediated by E-protein activity. Application of inhibitors would then oppose the cytotoxic effects of the E protein and restore viability (Figure 3 and Supplementary Figure 1).

Apigenin showed a biphasic effect on viability: small concentrations (<10 μM) increased viability, while higher concentrations (20–50 μM) were cytotoxic. Most flavonoids showed increased cell viability at concentrations between 20 and 50 μM. Rimantadine and HMA had IC_50_ values of 15.9 ± 3.5 and 2.4 ± 0.3 μM, respectively, for inhibition of E-protein induced cytotoxicity (Figure 3). We noted that HMA (hexamethylene amiloride) at concentrations larger than 10 μM induced cell death independent of E protein expression, so HMA could only be tested at low (<5 μM) concentrations. Although effective, the cytotoxicity of HMA may limit its therapeutic potential. The potency of amantadine as E-protein inhibitor was six-fold reduced compared to rimantadine with an IC_50_ of 77.3 ± 12.6 μM. In addition to the classical viroporin inhibitors we tested several flavonoids and two gingerols (Figure 3). Some flavonoids showed activities similar to that of rimantadine. Most potent compound was EGCG (IC_50_ = 2.9 ± 1.3 μM), while Nobiletin (IC_50_ = 51.1 ± 13 μM) had the lowest activity of the tested flavonoids. Gingerols 6- and 8-gingerol were less potent with half-maximum concentrations of 48 ± 7.3 μM and 69.0 ± 15.8 μM (Figure 3 and Supplementary Figure 1).

**FIGURE 4:**
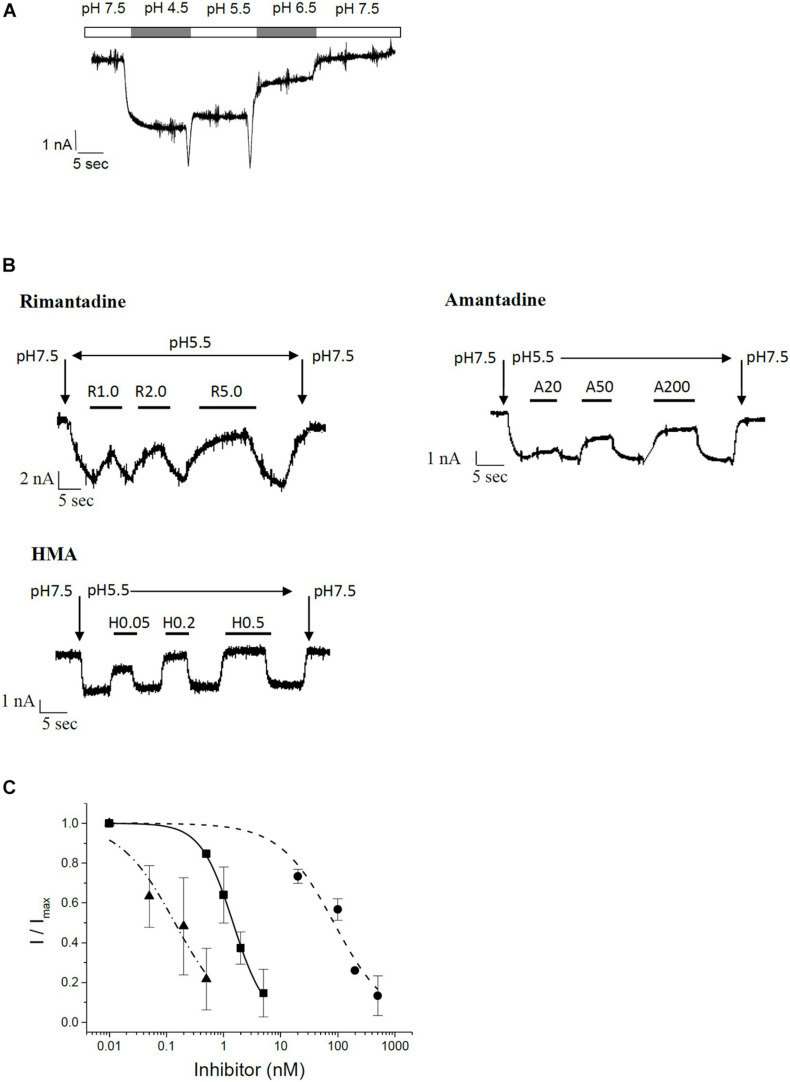
Patch clamp electrophysiology on recombinant SARS CoV E protein in HEK293 cells. **(A)** pH-dependent currents in permeant ion-minimized extracellular buffer (in mM: 90 N-Methyl-D-glucamine (NMDG), 3 CaCl_2_, 90 HEPES and 90 MES). Cells in the whole-cell configuration were perfused with buffer of the indicated pH, as indicated by bars above the trace. Here, lower pH defines a higher concentration of permeant protons, which leads to increased currents. **(B)** Inhibition of E channel-mediated currents by rimantadine, amantadine and HMA in minimized extracellular buffer. Baseline was measured at pH 7.5, reduction in pH to pH 5.5 resulted in an E protein-mediated inward current that could be inhibited by test compounds. At the end of each measurement, pH was switched back to control (7.5). Vertical arrows indicate switch to buffer with indicated pH. Bars denote flow application of indicated concentrations of inhibitor. **(C)** Summary of IC_50_ curves. Solid squares, solid line: rimantadine; solid circles, short-dashed line: amantadine; solid triangles, dash-dotted line: HMA.

To confirm the results obtained in the MTT assay, we studied channel function and inhibition of recombinant E protein using patch-clamp electrophysiological techniques. Investigation of E protein currents was performed in a special buffer where sodium and potassium were replaced by non-permeant NMDG, 3 mM CaCl_2_, HEPES and 2-(N-morpholino) ethane sulfonic acid (MES). The external buffer was adjusted to either pH 7.5 (mostly inactive) or pH 5.5 (active). The internal buffer contained NMDG, EGTA, and HEPES and was adjusted to pH 7.5. During measurements HEK293 cells were kept in classical extracellular buffer containing 135 mM NaCl, 5.5 mM KCl, 2 mM CaCl_2_, 1.0 mM MgCl_2_, and 10 mM Hepes. Only after patching and up-lifting they were permanently kept in a flow of NMDG-HEPES-MES buffer at different pH values. Under these specific conditions, H^+^ ions are the predominant permeant ion species carrying the inward currents. At low pH this current is pronounced, and reduced at pH 7.5 (Figure 4A). These proton currents can be blocked by specific channel inhibitors. We tested the classical viroporin inhibitors rimantadine, amantadine and HMA (Figure 4B). Under these conditions, HMA turned out to be the most potent inhibitor (IC_50_ = 0.14 ± 0.04 nM) followed by rimantadine (IC_50_ = 1.5 ± 0.1 nM), while amantadine (IC_50_ = 85 ± 19 nM) was less active (Figure 4C and Table 1).

**TABLE 1:** Inhibition of SARS-CoV E protein by classical viroporin inhibitors and flavonoids.

	MTT pRK control	MTT IC_50_ (μM)	MTT max. Inhibition (%)	patch clamp IC_50_ (nM)	IV ramp IC_50_ (nM)
**Classical**					
Rimantadine	neutral	15.9 ± 3.5	90	1.5 ± 0.1	1.7 ± 0.6
Amantadine	neutral	77.3 ± 12.6	82	85 ± 19	10.6 ± 0.5
HMA	10–50 ↓	2.4 ± 0.3	90	0.14 ± 0.04	0.27 ± 0.1
**Flavonoids**					
Apigenin	5–10↑ 20–50↓	6.6 ± 0.4	100		5.9 ± 0.1
EGCG	5–50 ↑	2.9 ± 1.3	100		1.54 ± 0.1
Genistein	20–50 ↑	9.9 ± 1.9	100		12.9 ± 0.7
Kaempferol	5–50 ↑	6.1 ± 2.1	100		11.5 ± 1.1
Naringenin	10–50 ↑	51.0 ± 8.8	75		*n.a.*
Nobiletin	5–50 ↑	55.1 ± 13.6	70		*n.a.*
Quercetin	10–50 ↑	2.0 ± 0.8	100		3.7 ± 0.2
Resveratrol	neutral	11.0 ± 0.7	90		4.7 ± 0.9
**Others**					
6-Gingerol	neutral	48 ± 7.3	80		*n.a.*
8-Gingerol	50 ↓	69.0 ± 15.8	60		*n.a.*

*MTT assays were performed on cells transfected with the empty pRK5 plasmid (pRK control), and with E-protein in pRK5 (IC_50_). Note that some inhibitors influenced cell growth: ↓ reduced cell growth in presence of inhibitor at given concentration; ↑ increased cell growth in presence of inhibitor at given concentration. For calculation of IC_50_, the effect of the inhibitor on cell growth was taken in account by normalization of IC_50_ values to pRK control. The MTT assay was used as a first test of inhibitory activity and followed by patch clamp recordings to confirm modulation of E protein channels. n.a. – not active.*

**FIGURE 5:**
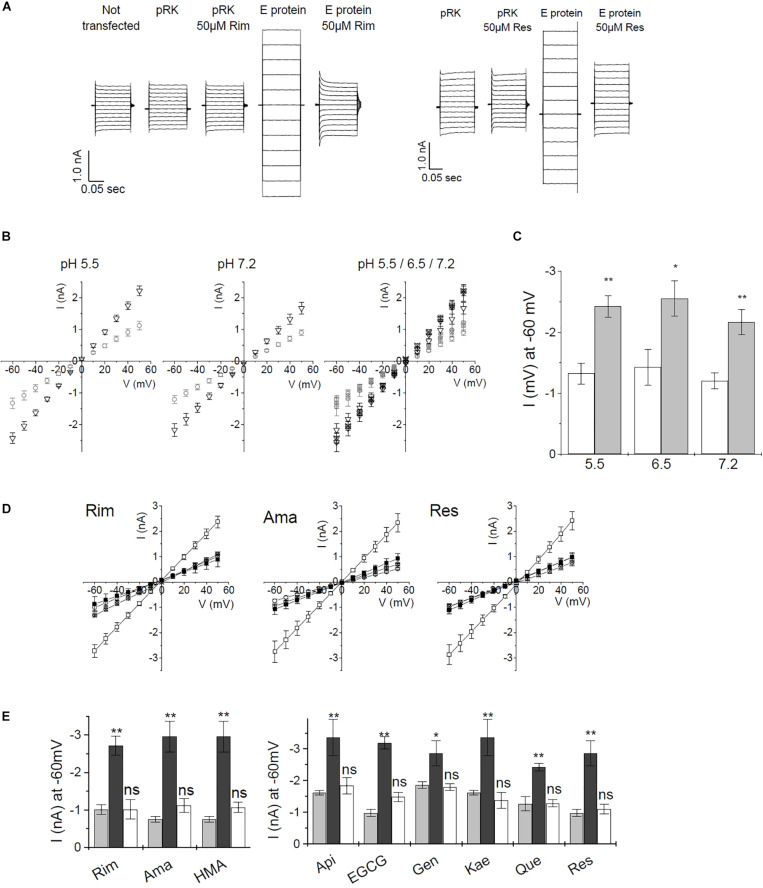
Current–voltage recordings from recombinant SARS CoV E protein in HEK293 cells. **(A)** Direct currents from a voltage ramp from –60 to +50 mV. Controls were (i) untransfected cells, (ii) mock (empty pRK vector) transfected HEK293 cells. Left panel: inhibition by rimantadine, right panel: inhibition by resveratrol. **(B)** pH dependence of control cells and E protein expressing HEK293 cells using standard extracellular buffer. Left and middle panel: open circles: control cells; open triangles: E protein expressing cells. Right panel: open symbols: pH 7.2; symbols including +: pH 6.5; crossed symbols: pH 5.5. Left and middle panel: pH 5.5 and 7.2, respectively. Right panel: summary of currents at pH 5.5, 6.5, and 7.2. Induced currents over the investigated pH range were not significantly different. **(C)** pH dependence of E-protein-mediated currents at –60 mV. White bars: pRK control; gray bars: currents induced after E protein expression. **(D)** Inhibition of E-protein channels by rimantadine, amantadine, and resveratrol. Open symbols: no inhibitor, solid symbols: in presence of 20 μM of inhibitor; circles: control (empty pRK) cells; squares: E protein expressing cells. **(E)** Summary of inhibition data. Light gray columns: control currents (pRK); dark gray columns: E-protein expressing cells; white columns: E-protein currents in the presence of inhibitor (20 μM). Significance was determined using one-way ANOVA with *p < 0.05; **p < 0.01, n.s. = not significant. See Supplementary Figure 2 for a complete set of inhibition data.

For whole-cell recordings, cells needed to be patched, detached and positioned in front of the perfusion device. At the time of measurement, a certain level of E protein expression had to be reached in order to generate a strong enough signal. After 2-day expression of E protein, we were not able to obtain channel-induced currents. When expression was allowed to proceed for 4 days or longer, cell damage due to E protein activity was so prominent that electrophysical measurements were not possible. The time window for successful measurements was small, even measurements from cells that expressed E protein for 3 days were difficult due to a large percentage of weakened cells which deteriorated during detaching and the subsequent applications of different buffer solutions (data not shown). For this reason, we changed the patch-clamp procedure to a technique where currents were recorded using classical external and internal buffers (see “Materials and Methods”). Under these conditions we could observe currents even at neutral pH of 7.2. Similar electrophysical measurements on HEK293 cells recombinantly expressing SARS CoV E protein were reported in the literature (Pervushin et al., 2009). Using this technique, the authors could show that HMA but not amiloride was blocking the E protein channel. The method allowed cells to remain attached to the cover slide throughout the measurement. Here, transfected cells were patched and the whole cell conformation established. After compensation, we measured IV ramps in the range of −60 to +50 mV (Figure 5A and Supplementary Figure 2), and determined channel activity from the slope conductance of the whole-dell currents. Inhibitors were applied to the bath solution, thus avoiding the necessity to lift cells from the coverslide. This faster and less stressful procedure allowed more efficient measurements, which is essential when screening inhibitors. We observed day to day variance for both, pRK (mock transfected), as well as E protein expressing HEK293 cells. Therefore, control recordings from mock- and E protein transfected cells were included in every experiment before testing inhibitors. All data were related to the control values of the same day.

To verify the method, we tested different pH conditions (pH 5.5, 6.5, and 7.2). Under all pH conditions, we observed similar current differences between mock-transfected control cells and E protein expressing cells. Differences in current amplitude were significant over the complete pH range (Figures 5B,C). These results indicated that the E protein channel is a general cation channel that is likely not proton-gated.

**FIGURE 6:**
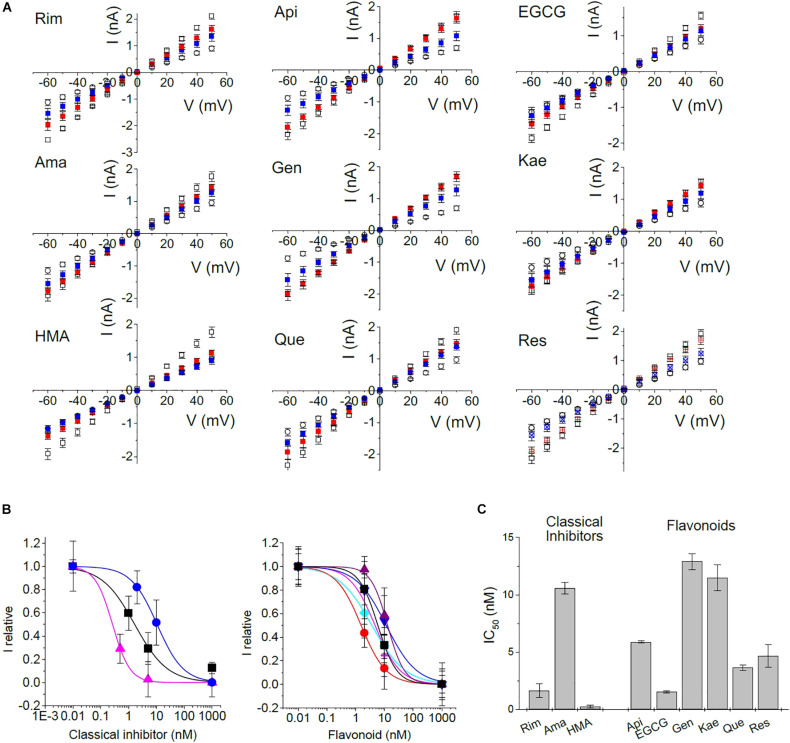
Concentration dependence of inhibition. To gain information about half-maximum inhibiting concentrations (IC_50_), IV-ramps were recorded at several concentrations of inhibitor. **(A)** Open circle: control currents (pRK5); open square: currents induced by E protein expression in the absence of inhibitor; red: currents at 2 nM of inhibitor, except Rim (1 nM) and HMA (0.5 nM); blue: currents at 10 nM of inhibitor except Rim and HMA (5 nM). **(B)** Concentration-response curves constructed from a non-linear fit of data shown in panel **(A)**. Left panel: black squares – Rim; blue circles – Ama; pink triangles – HMA; right panel: black square – Api; red circle – EGCG; purple up triangle – Gen; blue down triangle – Kae; turquoise diamond – Que; pink plus – Res. **(C)** Summary of IC_50_ values.

We then tested all inhibitors that had shown inhibition of E protein channels in the MTT assay using the voltage ramp patch-clamp protocol (Figures 5D,E and Supplementary Figure 2). Rimantadine, amantadine, HMA, as well as apigenin, EGCG, genistein, kaempferol, quercetin, and resveratrol showed robust channel blocking activity (Figure 5E and Supplementary Figure 2). To get more information on inhibitory potency, we tested two different concentrations for each inhibitor to get an estimate of half-maximum concentrations (Figure 6). Indeed, we observed inhibition at very low (nM range) concentrations of inhibitor, in good agreement with results obtained from single cell measurements in low Na^+^/K^+^ buffer (Figure 6C).

In patch-clamp recordings, we exclusively observed whole cell currents from E-protein channels that were correctly integrated into the plasma membrane. Activity of any intracellular E protein cannot be observed under these experimental conditions. For this reason, inhibitors do not have to enter the cell interior in order to be active, in contrast to most other cellular assays, including the MTT viability test. We observed estimated IC_50_ values of 1.7 ± 0.6, 10.6 ± 0.5, and 0.3 ± 0.1 nM for rimantadine, amantadine and HMA, respectively, in good agreement with data from low Na^+^/K^+^ buffers. Similar low IC_50_ values had been observed when cell surface p7 channels were studied by patch-clamp electrophysiology (Breitinger et al., 2016, 2021). Flavonoids yielded inhibition constants in the range of rimantadine with IC_50_ values ranging from 1.5 nM for EGCG and 12.9 nM for genistein. In addition to EGCG, quercetin also revealed high blocking activity (Table 1).

## Discussion

### Classical Inhibitors Amantadine, Rimantadine, and HMA

We confirmed the activity of the classical viroporin inhibitors amantadine, rimantadine, and HMA on SARS CoV in a cell viability assay and by patch clamp electrophysiology on recombinant E protein expressed in HEK293 cells. We found HMA the most active inhibitor followed by rimantadine. Amantadine acted as an inhibitor but activity was reduced six-fold (electrophysiology) to seven-fold (MTT viability assay) compared to rimantadine. Amantadine and HMA had been shown before to be inhibitors of the E protein of different Corona viruses, including SARS CoV (Wilson et al., 2006; Torres et al., 2007; Pervushin et al., 2009; Mandala et al., 2020). In 2004, it was shown that E protein from SARS CoV forms a cation-selective ion channel in planar lipid bilayers (Wilson et al., 2004). Later, the same group showed that envelope proteins from other CoVs form cation-selective ion channels, including the E protein from human coronavirus 229E (HCoV-229E), mouse hepatitis virus (MHV), and infectious bronchitis virus (IBV) (Wilson et al., 2006). HMA inhibits the HCoV-229E and MHV E protein ion channel conductance in bilayers and also inhibits replication of the parent CoVs in cultured cells (Wilson et al., 2006). Peptides corresponding to the transmembrane domain of SARS CoV E protein (residues 9–35), with two lysine residues flanking both N and C termini were reconstituted in lipid bilayers and investigated (Torres et al., 2007). Wild-type peptides were inhibited by amantadine, while the double mutants N15A-V25F and V24A-A32F were resistant against amantadine treatment (Torres et al., 2007). Using the patch clamp technique, current–voltage relationships show that HMA but not amiloride inhibits SARS CoV E protein after recombinant expression in HEK293 cells. The current–voltage relationship is compared to untransfected or vector transfected HEK293 cells (Pervushin et al., 2009). The same investigation localized two binding sites for HMA, one near N15, the second close to T35 and R38. Structure analysis was performed on the E protein transmembrane domain (ETM) using solution NMR in dodecyl-phosphatidylcholine micelles and energy minimization (Pervushin et al., 2009). A recent study on SARS CoV-2 ETM shows its structure in ERGIC-mimetic lipid bilayers bound to amantadine and HMA using NMR spectral analysis (Mandala et al., 2020). To our knowledge, rimantadine binding was not investigated on SARS CoV or CoV-2. Rimantadine is a well-known and -studied viroporin inhibitor, acting on influenza M2 (Fleming, 2001; Intharathep et al., 2008) and p7 channels of hepatitis C (OuYang et al., 2013; Breitinger et al., 2016).

### Flavonoids

Flavonoids are a group of natural substances with variable phenolic structures. They are mostly found in fruits, vegetables, grains, bark, roots, stems, tea, and red wine. Flavonoids are antioxidants that can reduce oxidative cellular stress. They are associated with numerous beneficial effects on human health including anticancer, anti-inflammatory and antiviral properties (Panche et al., 2016). Studies on the mechanisms that underlie the antiviral activity of flavonoids suggest a combination of effects on both, the virus and the host cell. Virus adsorption, entry, replication, viral protein translation, the formation of certain virus envelope glycoprotein complexes, and virus release was affected by flavonoids, as well as a variety of host cell signaling processes, including induction of gene transcription factors and secretion of cytokines (Wang et al., 2020). Traditional Chinese Medicine is based on natural compounds like flavonoids. The influence of flavonoids on SARS CoV-2 was discussed in several reviews (Bastaminejad and Bakhtiyari, 2020; Colunga Biancatelli et al., 2020; Huang et al., 2020; Russo et al., 2020; Solnier and Fladerer, 2020). Some flavonoids were described to inhibit key proteins involved in coronavirus infective cycle, such as the inflammatory mediators PLpro, 3CLpro, NTPase/^∗^/7 were mentioned as inhibitors of inflammatory mediators (Huang et al., 2020), while quercetin was reported to reduce the level of IL-6 (Bastaminejad and Bakhtiyari, 2020). Presence of intact E-protein is essential for virus replication, as demonstrated in a study comparing mice that were infected either with SARS CoV or with a mutated SARS CoV missing the E protein. Mice infected with the complete virus showed rapid loss of weight and subsequent death. In contrast, mice infected with the virus lacking the E protein mostly recovered from the disease and survived (Nieto-Torres et al., 2014). The same study showed that levels of inflammasome-activated IL-1β were reduced in the lung airways of the animals infected with viruses lacking E protein activity, indicating that not only the 3a protein (Siu et al., 2019), but also E protein ion channel activity is required for inflammasome activation, possibly serving as second trigger in a similar way as was suggested for p7 in hepatitis C (Shrivastava et al., 2013; Farag et al., 2017, 2020; Negash et al., 2019). Furthermore, reduction of IL-1β was accompanied by diminished amounts of TNF and IL-6 in the absence of E protein ion conductivity (Nieto-Torres et al., 2014). The expression of the SARS-CoV E protein is associated with a hyper-inflammatory response that could culminate in the ARDS of COVID-19 (Schoeman and Fielding, 2020). Essentially, this immune-mediated damage is caused by a cytokine storm, induced by significantly elevated levels of inflammatory cytokines IL-1β and IL-6 that are partly mediated by E protein expression. The interaction between the SARS-CoV E protein and the host protein – syntenin – as well as the viroporin function of SARS-CoV E, are linked to this cytokine dysregulation (Schoeman and Fielding, 2020). These observations would be in good agreement with our findings that some flavonoids – including quercetin – inhibit the E protein. It is noted that the E protein is highly conserved in the SARS family; only four out of 76 amino acid residues are different between the E proteins of SARS-CoV and SARS-CoV-2. These four exchanges cluster near the C terminus, adding a positive charge to this protein region, are not expected to affect the ion pore of the E protein channel. Thus, viroporin inhibitors may be active against the E protein of both coronavirus strains.

Several flavonoids were identified to inhibit members of the viroporin protein family. Genistein, as well as naringenin (to a lesser extent), but not quercetin, kaempferol, (–)epigallocatechin, or (–)epicatechin were able to inhibit viral protein U of human immunodeficiency type 1 virus when protein U was expressed heterologously in *Xenopus* oocytes and drug effects tested using voltage clamp experiments (Sauter et al., 2014). A study on heterologously expressed SARS CoV 3a protein in *Xenopus* oocytes tested the flavonols kaempferol, kaempferol glycosides, and kaempferol derivatives for activity against the 3a ion channel. Some 3a-inhibitory kaempferol derivatives were identified, the glycoside juglanin being the most efficient (Schwarz et al., 2014). A computational docking study of p7 ion channel from HCV Genotype 3 and Genotype 4 tested different flavonoids including Epigallocatechin-3-gallate, apigenin, naringenin, luteolin, quercetin, ladanein, silymarin, honokiol, and nobiletin, showing the existence of flavonoid binding pockets in different viroporins (Mathew et al., 2015). Our results identify quercetin and EGCG as potential ligands and inhibitors of the SARS-CoV E protein.

### pH Dependence of E Protein Channel Activity

When we investigated SARS CoV E protein currents under permeant ion-reduced extracellular buffer (replacing Na^+^ and K^+^ with N-methyl D-gluconate), we could record pH dependent currents which increased with decreasing pH, similar to observations with hepatitis C p7 channels (Breitinger et al., 2016). We concluded that this observation was due to increased amounts of the major permeant H^+^ ion at lower pH values. This was confirmed when measuring E protein at different pH conditions in standard extracellular buffer (in mM: 135 NaCl, 5.5 KCl, 2.0 CaCl_2_, 1.0 MgCl_2_, and 10 Hepes). Under these conditions, with no limitation on the concentration of permeant ions, no pH dependence of the E protein channel was detectable. Thus, proton conductance may be one aspect of E protein channel activity, but the role of the E protein in COVID pathology can also be accounted for by its activity as a general intracellular cation channel, similar to other viroporins.

## Conclusion

We have studied the E protein of the SARS-coronavirus using two independent techniques, patch-clamp electrophysiology and a cell viability assay. Known viroporin inhibitors as well as a series of flavonoids and gingerols were tested as potential inhibitors of E protein channel activity. The E protein is a cation channel that is inhibited by classical viroporin inhibitors amantadine, rimantadine, and HMA. Several flavonoids were shown to inhibit the channel with a potency similar to that of rimantadine, with EGCG showing the highest inhibitory activity. Our results confirm the channel activity of the E protein and suggest flavonoids and related substances as potential viroporin inhibitors and therapeutic leads to combat SARSCoV infections.

## Data Availability Statement

The raw data supporting the conclusions of this article will be made available by the authors, without undue reservation.

## Author Contributions

UB: conceptualization, methodology, validation, formal analysis, investigation, resources, writing (original draft, review, and editing), supervision, and project and administration. NA: formal analysis and investigation. HS: validation, investigation, and resources. H-GB: conceptualization, methodology, validation, formal analysis, investigation, resources, writing (original draft, review, and editing), supervision, and project administration. All authors contributed to the article and approved the submitted version.

## Conflict of Interest

The authors declare that the research was conducted in the absence of any commercial or financial relationships that could be construed as a potential conflict of interest.

## References

[B1] An S. Chen C. J. Yu X. Leibowitz J. L. Makino S. (1999). Induction of apoptosis in murine Coronavirus-infected cultured cells and demonstration of E protein as an apoptosis inducer. *J. Virol.* 73 7853–7859. 10.1128/jvi.73.9.7853-7859.1999 10438879 PMC104316

[B2] Andrew A. Strebel K. (2010). HIV-1 Vpu targets cell surface markers CD4 and BST-2 through distinct mechanisms. *Mol. Aspects Med.* 31 407–417. 10.1016/j.mam.2010.08.002 20858517 PMC2967615

[B3] Arbely E. Khattari Z. Brotons G. Akkawi M. Salditt T. Arkin I. T. (2004). A highly unusual palindromic transmembrane helical hairpin formed by SARS Coronavirus E protein. *J. Mol. Biol.* 341 769–77944. 10.1016/j.jmb.2004.06.044 15288785 PMC7134595

[B4] Bastaminejad S. Bakhtiyari S. (2020). Quercetin and its relative therapeutic potential against COVID-19: a retrospective review and prospective overview. *Curr. Mol. Med.* 10.2174/1566524020999200918150630 [Online ahead of print]. 32957884

[B5] Breitinger U. Farag N. S. Ali N. K. M. Ahmed M. El-Azizi M. A. Breitinger H. G. (2021). Cell viability assay as a tool to study activity and inhibition of hepatitis C p7 channels. *J. Gen. Virol.* 102:2086. 10.1099/jgv.0.001571 33709903

[B6] Breitinger U. Farag N. S. Ali N. K. Breitinger H. G. (2016). Patch-clamp study of Hepatitis C p7 channels reveals genotype-specific sensitivity to inhibitors. *Biophys. J.* 110 2419–2429. 10.1016/j.bpj.2016.04.018 27276260 PMC4906147

[B7] Castano-Rodriguez C. Honrubia J. M. Gutierrez-Alvarez J. DeDiego M. L. Nieto-Torres J. L. Jimenez-Guardeno J. L. (2018). Role of severe acute respiratory syndrome Coronavirus Viroporins E, 3a, and 8a in replication and pathogenesis. *mBio* 9:e02325-17. 10.1128/mBio.02325-17 PMC5964350 29789363

[B8] Chizhmakov I. V. Ogden D. C. Geraghty F. M. Hayhurst A. Skinner A. Betakova T. (2003). Differences in conductance of M2 proton channels of two influenza viruses at low and high pH. *J. Physiol.* 546(Pt 2), 427–438. 10.1113/jphysiol.2002.028910 12527729 PMC2342522

[B9] Colunga Biancatelli R. M. L. Berrill M. Catravas J. D. Marik P. E. (2020). Quercetin and Vitamin C: an experimental, synergistic therapy for the prevention and treatment of SARS-CoV-2 related disease (COVID-19). *Front. Immunol.* 11:1451. 10.3389/fimmu.2020.01451 PMC7318306 32636851

[B10] Costela-Ruiz V. J. Illescas-Montes R. Puerta-Puerta J. M. Ruiz C. Melguizo-Rodriguez L. (2020). SARS-CoV-2 infection: the role of cytokines in COVID-19 disease. *Cytokine Growth Factor Rev.* 54 62–75. 32513566 10.1016/j.cytogfr.2020.06.001 PMC7265853

[B11] DeDiego M. L. Nieto-Torres J. L. Jimenez-Guardeno J. M. Regla Nava J. A. Alvarez E. Oliveros J. C. (2011). Severe acute respiratory syndrome Coronavirus envelope protein regulates cell stress response and apoptosis. *PLoS Pathog.* 7:e1002315. 10.1371/journal.ppat.1002315 22028656 PMC3197621

[B12] Drosten C. Gunther S. Preiser W. van der Werf S. Brodt H. R. Becker S. (2003). Identification of a novel Coronavirus in patients with severe acute respiratory syndrome. *N. Engl. J. Med.* 348 1967–1976. 12690091 10.1056/NEJMoa030747

[B13] Duff K. C. Ashley R. H. (1992). The transmembrane domain of influenza A M2 protein forms amantadine-sensitive proton channels in planar lipid bilayers. *Virology* 190 485–489. 10.1016/0042-6822(92)91239-q 1382343

[B14] Ewart G. D. Sutherland T. Gage P. W. Cox G. B. (1996). The Vpu protein of human immunodeficiency virus type 1 forms cation-selective ion channels. *J. Virol.* 70 7108–7115. 10.1128/jvi.70.10.7108-7115.1996 8794357 PMC190763

[B15] Farag N. S. Breitinger U. Breitinger H. G. El Azizi M. A. (2020). Viroporins and inflammasomes: a key to understand virus-induced inflammation. *Int. J. Biochem. Cell Biol.* 122:105738. 10.1016/j.biocel.2020.105738 32156572 PMC7102644

[B16] Farag N. S. Breitinger U. El-Azizi M. Breitinger H. G. (2017). The p7 viroporin of the hepatitis C virus contributes to liver inflammation by stimulating production of Interleukin-1β. *Biochim. Biophys. Acta Mol. Basis Dis.* 1863 712–720. 10.1016/j.bbadis.2016.12.006 27979709

[B17] Fleming D. M. (2001). Managing influenza: amantadine, rimantadine and beyond. *Int. J. Clin. Pract.* 55 189–195. 11351773

[B18] Freeman T. L. Swartz T. H. (2020). Targeting the NLRP3 inflammasome in severe COVID-19. *Front. Immunol.* 11:1518. 10.3389/fimmu.2020.01518 PMC7324760 32655582

[B19] Gonzalez J. M. Gomez-Puertas P. Cavanagh D. Gorbalenya A. E. Enjuanes L. (2003). A comparative sequence analysis to revise the current taxonomy of the family Coronaviridae. *Arch. Virol.* 148 2207–2235. 10.1007/s00705-003-0162-1 14579179 PMC7087110

[B20] Griffin S. D. Beales L. P. Clarke D. S. Worsfold O. Evans S. D. Jaeger J. (2003). The p7 protein of hepatitis C virus forms an ion channel that is blocked by the antiviral drug, Amantadine. *FEBS Lett.* 535 34–38. 10.1016/s0014-5793(02)03851-6 12560074

[B21] Hover S. Foster B. Barr J. N. Mankouri J. (2017). Viral dependence on cellular ion channels - an emerging anti-viral target↑ *J. Gen. Virol.* 98 345–351. 10.1099/jgv.0.000712 28113044

[B22] Huang Y. F. Bai C. He F. Xie Y. Zhou H. (2020). Review on the potential action mechanisms of Chinese medicines in treating Coronavirus disease 2019 (COVID-19). *Pharmacol. Res.* 158:104939. 10.1016/j.phrs.2020.104939 32445956 PMC7239792

[B23] Intharathep P. Laohpongspaisan C. Rungrotmongkol T. Loisruangsin A. Malaisree M. Decha P. (2008). How amantadine and rimantadine inhibit proton transport in the M2 protein channel. *J. Mol. Graph. Model.* 27 342–348. 10.1016/j.jmgm.2008.06.002 18620883

[B24] Jalily P. H. Eldstrom J. Miller S. C. Kwan D. C. Tai S. S. Chou D. (2016). Mechanisms of action of novel influenza A/M2 viroporin inhibitors derived from hexamethylene amiloride. *Mol. Pharmacol.* 90 80–95. 10.1124/mol.115.102731 27193582

[B25] Jing X. Ma C. Ohigashi Y. Oliveira F. A. Jardetzky T. S. Pinto L. H. (2008). Functional studies indicate amantadine binds to the pore of the influenza a virus M2 proton-selective ion channel. *Proc. Natl. Acad. Sci. U. S. A.* 105 10967–10972. 10.1073/pnas.0804958105 18669647 PMC2492755

[B26] Li Y. Surya W. Claudine S. Torres J. (2014). Structure of a conserved Golgi complex-targeting signal in Coronavirus envelope proteins. *J. Biol. Chem.* 289 12535–12549. 10.1074/jbc.m114.560094 24668816 PMC4007446

[B27] Liao Y. Yuan Q. Torres J. Tam J. P. Liu D. X. (2006). Biochemical and functional characterization of the membrane association and membrane permeabilizing activity of the severe acute respiratory syndrome Coronavirus envelope protein. *Virology* 349 264–275. 10.1016/j.virol.2006.01.028 16507314 PMC7111751

[B28] Liu J. Xie W. Wang Y. Xiong Y. Chen S. Han J. (2020). A comparative overview of COVID-19, MERS and SARS: review article. *Int. J. Surg.* 81 1–8. 10.1016/j.ijsu.2020.07.032 32730205 PMC7382925

[B29] Lu W. Zheng B. J. Xu K. Schwarz W. Du L. Wong C. K. (2006). Severe acute respiratory syndrome-associated Coronavirus 3a protein forms an ion channel and modulates virus release. *Proc. Natl. Acad. Sci. U. S. A.* 103 12540–12545. 10.1073/pnas.0605402103 16894145 PMC1567914

[B30] Mandala V. S. McKay M. J. Shcherbakov A. A. Dregni A. J. Kolocouris A. Hong M. (2020). Structure and drug binding of the SARS-CoV-2 envelope protein transmembrane domain in lipid bilayers. *Nat. Struct. Mol. Biol.* 27 1202–1208. 10.1038/s41594-020-00536-8 33177698 PMC7718435

[B31] Marra M. A. Jones S. J. Astell C. R. Holt R. A. Brooks-Wilson A. Butterfield Y. S. (2003). The genome sequence of the SARS-associated Coronavirus. *Science* 300 1399–1404. 10.1126/science.1085953 12730501

[B32] Mathew S. Fatima K. Fatmi M. Q. Archunan G. Ilyas M. Begum N. (2015). Computational docking study of p7 ion channel from HCV Genotype 3 and Genotype 4 and its interaction with natural compounds. *PLoS One* 10:e0126510. 10.1371/journal.pone.0126510 26030803 PMC4451521

[B33] Nal B. Chan C. Kien F. Siu L. Tse J. Chu K. (2005). Differential maturation and subcellular localization of severe acute respiratory syndrome Coronavirus surface proteins S, M and E. *J. Gen. Virol.* 86(Pt 5), 1423–1434. 10.1099/vir.0.80671-0 15831954

[B34] Negash A. A. Olson R. M. Griffin S. Gale M. (2019). Modulation of calcium signaling pathway by hepatitis C virus core protein stimulates NLRP3 inflammasome activation. *PLoS Pathog.* 15:e1007593. 10.1371/journal.ppat.1007593 30811485 PMC6392285

[B35] Nieto-Torres J. L. Dediego M. L. Alvarez E. Jimenez-Guardeno J. M. Regla-Nava J. A. Llorente M. (2011). Subcellular location and topology of severe acute respiratory syndrome Coronavirus envelope protein. *Virology* 415 69–82. 10.1016/j.virol.2011.03.029 21524776 PMC4726981

[B36] Nieto-Torres J. L. DeDiego M. L. Verdia-Baguena C. Jimenez-Guardeno J. M. Regla-Nava J. A. Fernandez-Delgado R. (2014). Severe acute respiratory syndrome Coronavirus envelope protein ion channel activity promotes virus fitness and pathogenesis. *PLoS Pathog.* 10:e1004077. 10.1371/journal.ppat.1004077 24788150 PMC4006877

[B37] Nieto-Torres J. L. Verdia-Baguena C. Jimenez-Guardeno J. M. Regla-Nava J. A. Castano-Rodriguez C. Fernandez-Delgado R. (2015). Severe acute respiratory syndrome Coronavirus E protein transports calcium ions and activates the NLRP3 inflammasome. *Virology* 485 330–339. 10.1016/j.virol.2015.08.010 26331680 PMC4619128

[B38] Nieva J. L. Madan V. Carrasco L. (2012). Viroporins: structure and biological functions. *Nat. Rev. Microbiol.* 10 563–574. 10.1038/nrmicro2820 22751485 PMC7097105

[B39] OuYang B. Xie S. Berardi M. J. Zhao X. Dev J. Yu W. (2013). Unusual architecture of the p7 channel from hepatitis C virus. *Nature* 498 521–525. 10.1038/nature12283 23739335 PMC3725310

[B40] Panche A. N. Diwan A. D. Chandra S. R. (2016). Flavonoids: an overview. *J. Nutr. Sci.* 5:e47. 10.1017/jns.2016.41 PMC5465813 28620474

[B41] Pavlovic D. Neville D. C. Argaud O. Blumberg B. Dwek R. A. Fischer W. B. (2003). The hepatitis C virus p7 protein forms an ion channel that is inhibited by long-alkyl-chain iminosugar derivatives. *Proc. Natl. Acad. Sci. U. S. A.* 100 6104–6108. 10.1073/pnas.1031527100 12719519 PMC156333

[B42] Pervushin K. Tan E. Parthasarathy K. Lin X. Jiang F. L. Yu D. (2009). Structure and inhibition of the SARS Coronavirus envelope protein ion channel. *PLoS Pathog.* 5:e1000511. 10.1371/journal.ppat.1000511 19593379 PMC2702000

[B43] Pinto L. H. Holsinger L. J. Lamb R. A. (1992). Influenza virus M2 protein has ion channel activity. *Cell* 69 517–528. 10.1016/0092-8674(92)90452-i 1374685

[B44] Ruch T. R. Machamer C. E. (2012). The Coronavirus E protein: assembly and beyond. *Viruses* 4 363–382. 10.3390/v4030363 22590676 PMC3347032

[B45] Russo M. Moccia S. Spagnuolo C. Tedesco I. Russo G. L. (2020). Roles of flavonoids against Coronavirus infection. *Chem. Biol. Interact.* 328:109211. 10.1016/j.cbi.2020.109211 32735799 PMC7385538

[B46] Sauter D. Schwarz S. Wang K. Zhang R. Sun B. Schwarz W. (2014). Genistein as antiviral drug against HIV ion channel. *Planta Med.* 80 682–687. 10.1055/s-0034-1368583 24963618

[B47] Schoeman D. Fielding B. C. (2020). Is there a link between the pathogenic human coronavirus envelope protein and immunopathology? A review of the literature. *Front. Microbiol.* 11:2086. 10.3389/fmicb.2020.02086 33013759 PMC7496634

[B48] Schwarz S. Sauter D. Wang K. Zhang R. Sun B. Karioti A. (2014). Kaempferol derivatives as antiviral drugs against the 3a channel protein of Coronavirus. *Planta Med.* 80 177–182. 10.1055/s-0033-1360277 24458263 PMC7171712

[B49] Scott C. Griffin S. (2015). Viroporins: structure, function and potential as antiviral targets. *J. Gen. Virol.* 96 2000–2027. 10.1099/vir.0.000201 26023149

[B50] Shrivastava S. Mukherjee A. Ray R. Ray R. B. (2013). Hepatitis C virus induces interleukin-1beta (IL-1beta)/IL-18 in circulatory and resident liver macrophages. *J. Virol.* 87 12284–12290. 10.1128/jvi.01962-13 24006444 PMC3807883

[B51] Siu K. L. Yuen K. S. Castano-Rodriguez C. Ye Z. W. Yeung M. L. Fung S. Y. (2019). Severe acute respiratory syndrome Coronavirus ORF3a protein activates the NLRP3 inflammasome by promoting TRAF3-dependent ubiquitination of ASC. *FASEB J.* 33 8865–8877. 10.1096/fj.201802418r 31034780 PMC6662968

[B52] Solnier J. Fladerer J. P. (2020). Flavonoids: a complementary approach to conventional therapy of COVID-19↑ *Phytochem. Rev.* 10.1007/s11101-020-09720-9726 Online ahead of print. PMC7500502 32982616

[B53] Surya W. Li Y. Torres J. (2018). Structural model of the SARS Coronavirus E channel in LMPG micelles. *Biochim. Biophys. Acta Biomembr.* 1860 1309–1317. 10.1016/j.bbamem.2018.02.017 29474890 PMC7094280

[B54] Torres J. Maheswari U. Parthasarathy K. Ng L. Liu D. X. Gong X. (2007). Conductance and amantadine binding of a pore formed by a lysine-flanked transmembrane domain of SARS Coronavirus envelope protein. *Protein Sci.* 16 2065–2071. 10.1110/ps.062730007 17766393 PMC2206980

[B55] Torres J. Parthasarathy K. Lin X. Saravanan R. Kukol A. Liu D. X. (2006). Model of a putative pore: the pentameric alpha-helical bundle of SARS Coronavirus E protein in lipid bilayers. *Biophys. J.* 91 938–947. 10.1529/biophysj.105.080119 16698774 PMC1563757

[B56] Verdia-Baguena C. Nieto-Torres J. L. Alcaraz A. DeDiego M. L. Torres J. Aguilella V. M. (2012). Coronavirus E protein forms ion channels with functionally and structurally-involved membrane lipids. *Virology* 432 485–494. 10.1016/j.virol.2012.07.005 22832120 PMC3438407

[B57] Wang L. Song J. Liu A. Xiao B. Li S. Wen Z. (2020). Research progress of the antiviral bioactivities of natural flavonoids. *Nat. Prod. Bioprospect.* 10 271–283. 10.1007/s13659-020-00257-x 32948973 PMC7500501

[B58] Wilson L. Gage P. Ewart G. (2006). Hexamethylene amiloride blocks E protein ion channels and inhibits Coronavirus replication. *Virology* 353 294–306. 10.1016/j.virol.2006.05.028 16815524 PMC7111787

[B59] Wilson L. McKinlay C. Gage P. Ewart G. (2004). SARS Coronavirus E protein forms cation-selective ion channels. *Virology* 330 322–331. 10.1016/j.virol.2004.09.033 15527857 PMC7111769

[B60] Zhang K. Hou Q. Zhong Z. Li X. Chen H. Li W. (2013). Porcine reproductive and respiratory syndrome virus activates inflammasomes of porcine alveolar macrophages via its small envelope protein E. *Virology* 442 156–162. 10.1016/j.virol.2013.04.007 23664331

