# Supplementary material for: Inhibition of SARS CoV Envelope Protein by Flavonoids and Classical Viroporin Inhibitors
Source: Front Microbiol. 2021 Jul 8;12:692423. doi: 10.3389/fmicb.2021.692423 (PMC8297954; doi:10.3389/fmicb.2021.692423)
Supplement: Supplementary file 1 [file Data_Sheet_1.PDF]

Supplementary Figures

Figure S1

A

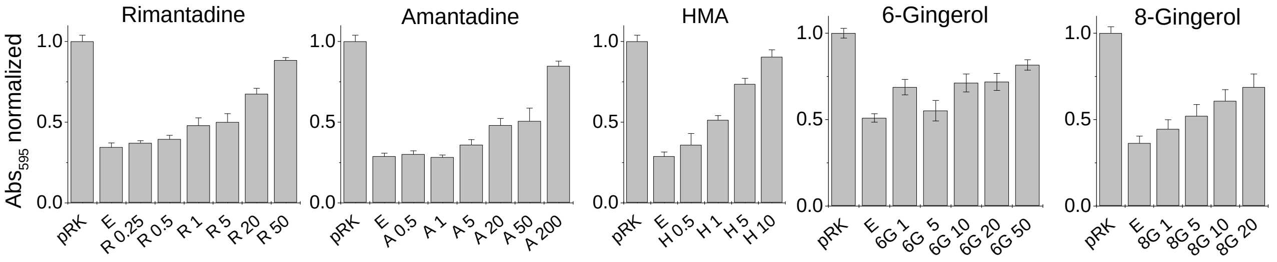

B

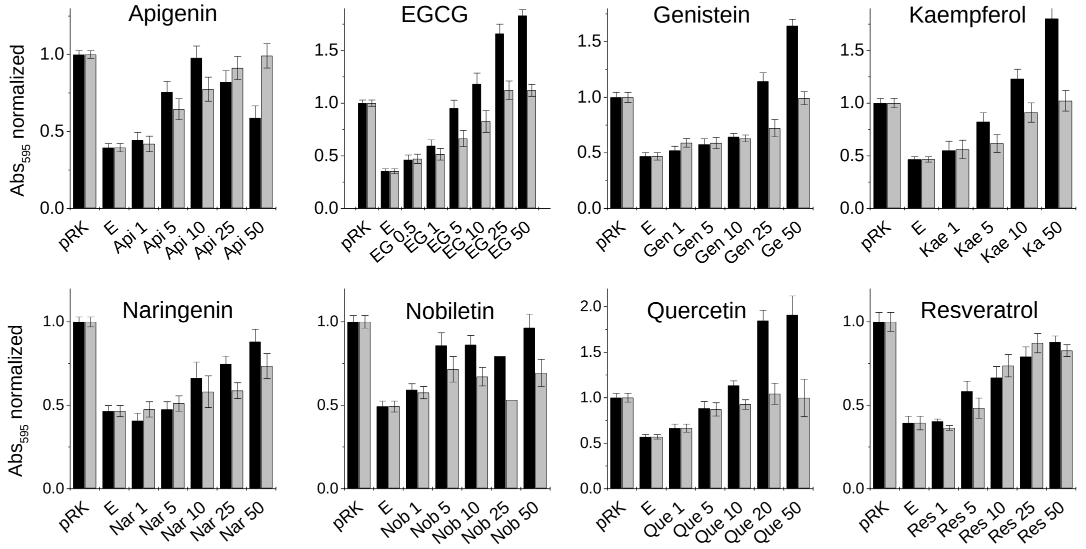

C

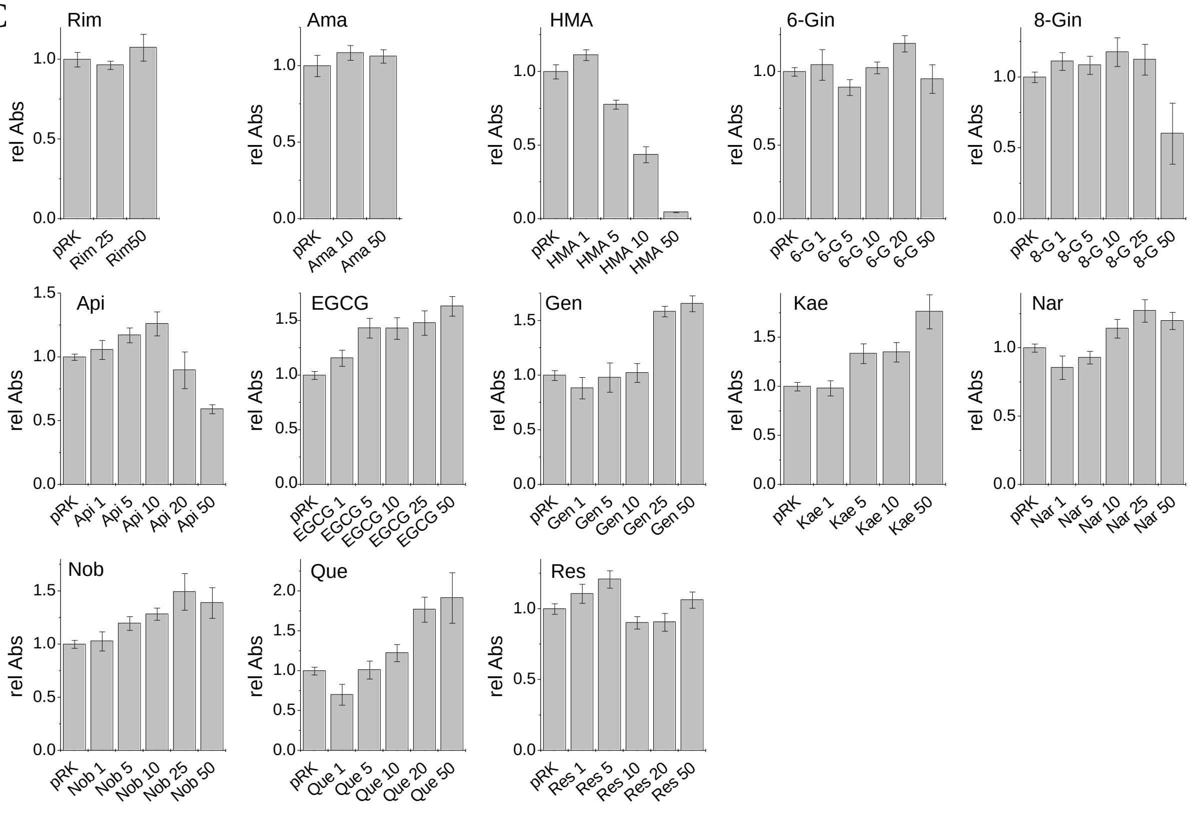

Figure S2

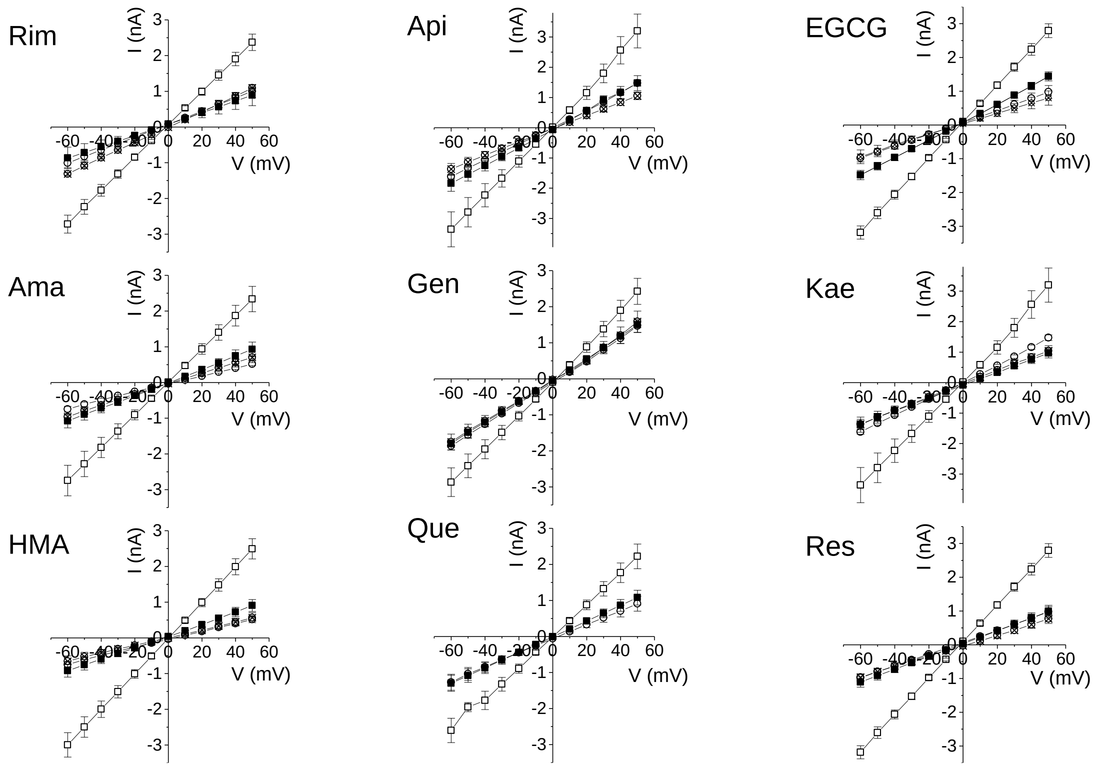

Supplementary Figure Legends

Figure S1 MTT Assay data.

(A) Normalized absorbance was obtained by first subtracting background (complete cell death induced by 200 mM KCl) and then dividing by control absorbance (pRK transfected HEK293 cells) HMA induced cell death at concentrations >10 µM, thus, higher concentrations were ignored. (B) Several flavonoids induced increased cell growth. Black columns show cell viability of E protein expressing HEK293 cells in presence of varying concentrations of inhibitor. Gray columns show the corrected signal, obtained after dividing by the pRK-vector control under same inhibitor concentration. (C) Cell viability data of control cells (transfected with empty pRK5) in presence of varying concentrations of inhibitor. Control experiments reveal cytotoxic (eg HMA, apigenin) and proliferative (eg genistein, quercetin, kaempferol) effects at increasing concentrations of E protein inhibitors.

Figure S2 Current-voltage relations of all investigated inhibitors in the absence and presence of 20 µM of inhibitor.

Open symbols: no inhibitor, solid symbols: 20 µM of inhibitor; circles: control (pRK); squares: E protein expressing cells.
